# Successful Management of Obstructive Fibrinous Tracheal Pseudomembrane with a Coring Technique after Preoxygenation with a High-Flow Nasal Cannula: A Case Report

**DOI:** 10.70352/scrj.cr.25-0330

**Published:** 2025-10-17

**Authors:** Takafumi Iguchi, Kensuke Kojima, Shuhei Kobayashi, Daiki Hayashi, Toshiteru Tokunaga, Hyungeun Yoon

**Affiliations:** Department of General Thoracic Surgery, NHO Kinki Chuo Chest Medical Center, Sakai, Osaka, Japan

**Keywords:** airway obstruction, fiberoptic intubation, high-flow nasal cannula, obstructive fibrinous tracheal pseudomembrane, post-intubation complication

## Abstract

**INTRODUCTION:**

Obstructive fibrinous tracheal pseudomembrane, an extremely rare but potentially fatal complication of tracheal intubation, occurs several days after intubation and is characterized by central airway obstruction due to the formation of a fibrinous pseudomembrane. Early diagnosis is crucial. Treatment typically involves bronchoscopic removal of the pseudomembrane. Herein, we describe a patient whose pseudomembrane was successfully cored out using fiberoptic intubation after achieving preoxygenation with high-flow nasal cannula oxygen therapy.

**CASE PRESENTATION:**

An 83-year-old woman (height 146 cm, weight 38 kg) underwent left lower lobe lobectomy with left atrial resection for squamous cell carcinoma (cT4N1M0, Stage IIIA). The surgery was completed successfully with no immediate complications. On POD 3, she developed prolonged expiration and inspiratory wheezing that was unresponsive to steroid inhalation. Her condition progressively worsened with stridor, labored breathing, and inability to lie supine. Bronchoscopy revealed 90% circumferential subglottic stenosis. High-flow nasal cannula oxygen therapy significantly improved her respiratory distress, enabling her to lie supine. Fiberoptic intubation was then performed under conscious sedation with spontaneous breathing, and the circumferential membranous structure was cored out by the intubation tube. Pathological examination confirmed fibrinous exudate with atypical cells, establishing a diagnosis of obstructive fibrinous tracheal pseudomembrane.

**CONCLUSIONS:**

Obstructive fibrinous tracheal pseudomembrane, a rare condition, causes upper airway obstruction within days after extubation. Preoxygenation with high-flow nasal cannula oxygen therapy, followed by coring the pseudomembrane out with an intubation tube, may be an effective, rapid, and minimally invasive means of treatment in selected patients with a subglottic, obstructive, fibrinous tracheal pseudomembrane.

## Abbreviations

FEV1
: forced expiratory volume in 1 s
FVC
: forced vital capacity
HFNC
: high-flow nasal cannula
OFTP
: obstructive fibrinous tracheal pseudomembrane

## INTRODUCTION

OFTP is a very rare, potentially fatal post-intubation complication that occurs several days after tracheal intubation and causes central airway obstruction due to the formation of a fibrinous pseudomembrane.^1,2)^ While removal of the pseudomembrane via a flexible or rigid bronchoscope is the standard treatment for OFTP, there have been few reports of coring the pseudomembrane out with an intubation tube. Here, we report safely coring out a pseudomembrane by performing fiberoptic intubation after preoxygenation with HFNC oxygen therapy.

## CASE PRESENTATION

**Fig. 1:**
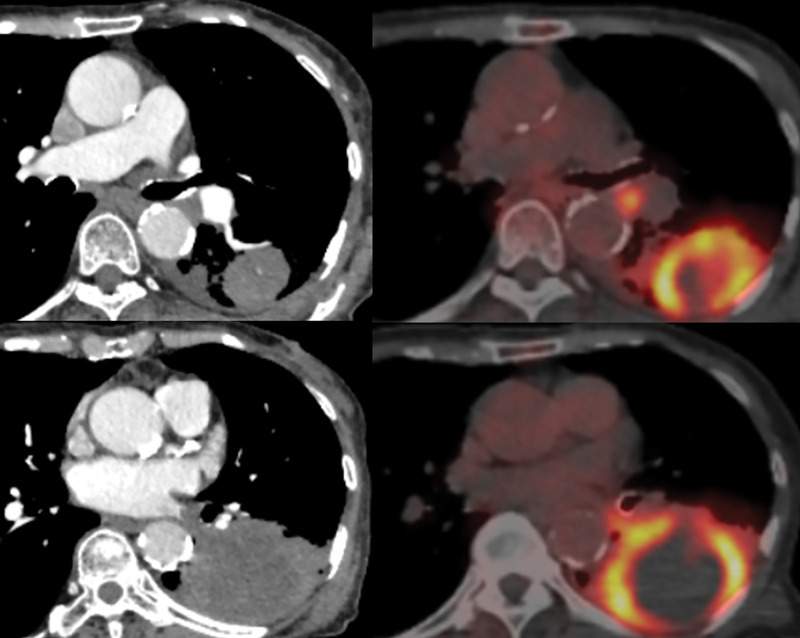
CT and PET images showing a large tumor in the left lower lobe of the lung and enlarged hilar lymph nodes with strong uptake of fluorodeoxyglucose. Tumor invasion of the left atrium is evident.

An 83-year-old woman (height 146 cm, weight 38 kg) was referred to our hospital for evaluation of a large left lung tumor that had been discovered by CT while investigating a productive cough. She had quit smoking 20 years previously after a 20-pack-year history. Her medical history included high blood pressure, lumbar compression fractures, and a distal gastrectomy for gastric cancer 20 years earlier. She walked with a cane but had an adequate performance status. No hoarseness or dyspnea was noted. A chest radiograph showed decreased transparency in the left middle and lower lung fields. A contrast CT scan showed a 78 mm mass in the left lower lobe and enlarged hilar lymph nodes (**Fig. 1**). The mass had invaded the lumen of the left inferior pulmonary vein, almost reaching the left atrium. Transbronchial tumor biopsy provided a pathological diagnosis of squamous cell carcinoma (no driver-gene alteration; proportion of tumor expressing programmed death-ligand 1, <1%). Whole-body CT and fluorodeoxyglucose-PET revealed no distant metastases but showed abnormal accumulation of standardized uptake value in the tumor and hilar lymph nodes (**Fig. 1**). She was diagnosed with left lower lobe squamous cell carcinoma, cT4N1M0, Stage IIIA.

Preoperative respiratory function tests showed no abnormalities (FVC, 2040 mL; percentage of the predicted FVC, 109%; FEV1, 1460 mL; percentage of the predicted value of FEV1, 94.2%; FEV1/FVC, 71.6%). An electrocardiogram showed a pulse rate of 95/min, sinus rhythm, and no ST-T changes. Transthoracic echocardiography revealed an ejection fraction of 58% and absence of regional wall motion abnormalities; all other measured variables were normal. Based on these findings, we decided to perform surgery.

Induction of anesthesia was uneventful. However, it proved impossible to pass a size 35 left double-lumen endotracheal tube through the glottis, necessitating the use of a size 32 left double-lumen endotracheal tube for intubation. Through a posterolateral thoracotomy, a left lower lobe lobectomy with resection of the left atrium was performed for a polypoidal tumor that was invading the intrapericardial inferior pulmonary vein as detailed below. There were no intrathoracic adhesions or extranodal invasion by the hilar lymph nodes. The left lower lobe pulmonary artery and bronchus were dissected with staplers, and the inferior pulmonary vein was secured outside the pericardium. The pericardium was then incised, and the border of the tumor was confirmed to have invaded the inferior pulmonary vein. After clamping the left atrium, the left atrial segment was excised and the defect closed with a continuous 5-0 prolene suture (Ethicon, Somerville, NJ, USA), whereas the pericardium was directly closed with a 4-0 polydioxanone suture (Ethicon), completing a left lower lobe lobectomy with left atrial resection (operation time 343 min, blood loss 620 mL, no blood transfusion required). The patient was extubated with a face mask in the operating room, after which she was transferred to the recovery room in a stable respiratory state without hoarseness or stridor.

**Fig. 2:**
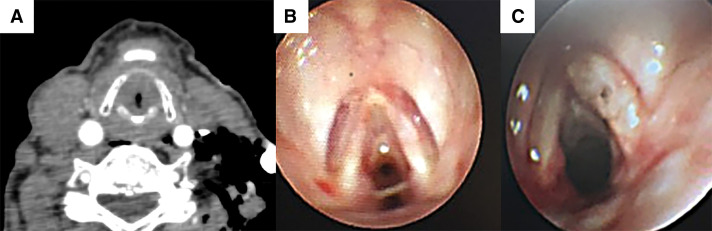
(**A**, **B**) Cervical CT and bronchoscopy images obtained before coring out show a circumferential membranous structure below the glottis that caused 90% stenosis. (**C**) Bronchoscopy images obtained the day after coring out show no remnants of the pseudomembrane, and the stenosis has been resolved.

**Fig. 3:**
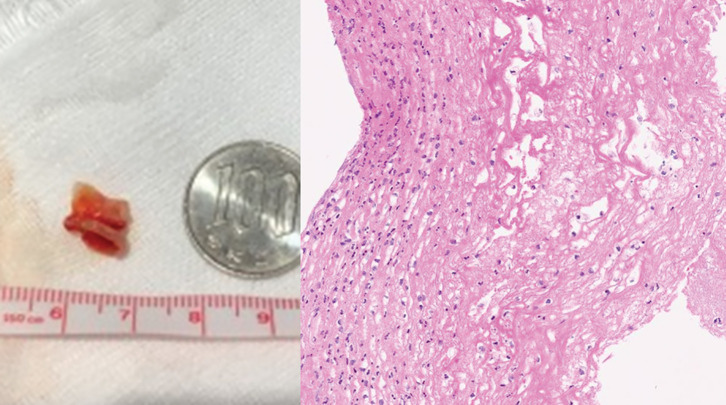
Left panel: Photograph of the removed cylindrical fleshy membrane. Right panel: Photomicrograph showing fibrinous exudate with inflammatory cells (hematoxylin and eosin stain, ×200).

**Fig. 4:**
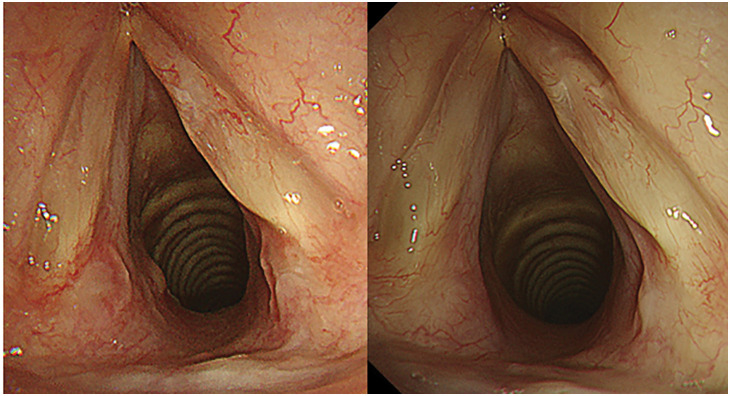
Bronchoscopy images obtained 2 weeks (left panel) and 4 weeks (right panel) after removal of the pseudomembrane, showing that the airway epithelium had healed well and there is no evidence of recurrence.

The patient’s postoperative course was uneventful until POD 2. However, on POD 3, although SpO_2_ remained stable at 96%, prolonged exhalation and wheezing during breathing were observed (PaO_2_ 82 torr, PaCO_2_ 45 torr). Steroid inhalation therapy was initiated but proved to be ineffective. The following day, the patient reported nausea accompanied by cold sweats and labored breathing. A chest radiograph showed no abnormalities. However, an electrocardiogram showed negative T waves in the chest leads that had not been present preoperatively. Blood biochemistry tests showed significant increases in myocardial enzyme levels and coagulation abnormalities (Troponin-I 535.6 pg/mL, D-dimer 4.9 μg/mL). Coronary angiography and contrast CT, performed because we suspected myocardial ischemia and pulmonary thromboembolism, showed only minor changes that were consistent with surgical manipulation. Her respiratory condition worsened progressively, stridor developed, and she had difficulty lying in a supine position. Although the patient’s SpO_2_ remained above 92%, her breathing pattern suggested central airway obstruction. Bedside bronchoscopy and CT revealed a circumferential 90% stenosis in the subglottis that a bronchoscope could not traverse (**Fig. 2**). The cause of tracheal stenosis was a membranous structure present circumferentially around the trachea. HFNC oxygen therapy (Optiflow; Fisher & Paykel Healthcare, Auckland, NZ) was commenced (flow rate 60 L/min, 100% FiO_2_, at 37°C), rapidly achieving improvement in the patient’s labored breathing and respiratory distress, which in turn enabled her to lie supine. Sufficient preoxygenation and stabilization of respiratory status were achieved with HFNC, allowing us to conclude that tracheal intubation could be safely performed concurrently with the coring out of the membrane. We decided to proceed with fiberoptic intubation due to its ability to increase the rigidity of the intubation tube, enable visualization of the membrane, and facilitate rapid removal of the membrane after coring out. Fiberoptic intubation was started with the patient breathing spontaneously under conscious sedation. This enabled coring out and removing the circumferential membranous structure found at the site of subglottic stenosis with an intubation tube, Shiley TaperGuard reinforced tracheal tube, 7.0 mm (Medtronic, Minneapolis, MN, USA) (**Fig. 3**). The patient was intubated overnight and extubated the following day, the subglottic stenosis having improved and the patient’s dyspnea and hoarseness resolved (**Fig. 2**). Pathological examination of the removed membranous structure revealed fibrinous exudate with some atypical cells (**Fig. 3**). The patient was diagnosed with OFTP. Her progress thereafter was uneventful, and she was discharged home 2 weeks after surgery. Bronchoscopy 2 and 4 weeks after coring out confirmed good healing of the bronchial epithelium (**Fig. 4**).

## DISCUSSION

Central airway obstruction caused by OFTP can rapidly prove fatal.^1–3)^ Early diagnosis by bronchoscopy is therefore essential.^1–3)^ OFTP is typically triggered by epithelial injury during intubation and most commonly occurs in the subglottic region.^3)^ Although symptoms usually appear within 24 h of extubation, they are often nonspecific—such as cough and hoarseness—resulting in diagnostic delays of 6–72 h.^3)^ In the present case, the patient manifested dyspnea with cold sweats on POD 3, raising concern about cardiovascular complications of left atrial resection. Initial evaluations were therefore mainly focused on possible myocardial ischemia and thromboembolic events. However, urgent bronchoscopy prompted by progression of her symptoms revealed subglottic obstruction due to OFTP. Given that airway obstruction can deteriorate more rapidly than cardiovascular events, early airway assessment should be prioritized in similar clinical scenarios.

Treatment of OFTP most commonly involves removal of the pseudomembrane via rigid or flexible bronchoscopy.^1)^ Some reports advocate performing a tracheotomy for airway management followed by bronchoscopic removal of the pseudomembrane in patients with subglottic OFTP.^4–6)^ However, 12.8% of adults with OFTP reportedly expectorate the pseudomembrane spontaneously within several days of extubation,^3)^ indicating that these pseudomembranes are extremely fragile and only loosely connected with the tracheal epithelium; they can thus be easily removed. In our case, HFNC improved oxygenation and reduced respiratory distress to the extent that the patient could tolerate the supine position, enabling safe fiberoptic-guided intubation under conscious sedation. The intubation tube itself cored out the pseudomembrane. If HFNC had not enabled adequate oxygenation and positioning, we would have considered performing an emergency tracheotomy. The utility of HFNC for preoxygenation, particularly prior to fiberoptic intubation, is well-supported in recent published reports.^7)^ Indeed, one previous report described unintentional coring out by an intubation tube that led to both diagnosis and treatment of OFTP.^8)^ While keeping the possibility of emergency tracheostomy in mind, preoxygenation with HFNC and coring out of subglottic OFTP with an intubation tube provides both rapidity and minimal invasiveness.

## CONCLUSIONS

The rare condition OFTP should be recognized as a cause of upper airway obstruction within several days of extubation. Our case indicates that removal of the pseudomembrane via an intubation tube after HFNC therapy can be an effective approach to treating subglottic OFTP.

## DECLARATIONS

### Availability of data and materials

The datasets supporting the conclusions of this article are included within the article.
